# Research on the Foaming Characteristics and Rheological Properties of Warm-Mix Foamed Polymer-Modified Bitumen Based on Waste Molecular Sieves

**DOI:** 10.3390/polym17182516

**Published:** 2025-09-17

**Authors:** Qiang Ye, Gongying Ding, Meng Yuan, Bei Chen

**Affiliations:** 1 The Second Engineering Company of CCCC Fourth Harbor Engineering Co., Ltd., Guangzhou 510230, China; qiangye2025@126.com; 2 College of Civil Engineering and Architecture, Wuyi University, Jiangmen 529020, China; 3 CCCC Fourth Harbor Engineering Co., Ltd., Guangzhou 510290, China; mengyuan202509@126.com; 4 School of Water Conservancy and Transportation, Zhengzhou University, Zhengzhou 450001, China

**Keywords:** polymer-modified bitumen, waste molecular sieve, foaming characteristics, high temperature rheological properties

## Abstract

Warm-mix foamed polyurethane modified bitumen (WPB) has been widely promoted due to its significant warm-mix effect and high viscosity. However, it still has problems such as too fast foam dissipation and unstable performance. Waste molecular sieves have an extremely fine pore structure that can absorb moisture. The porous characteristics of waste molecular sieves are used to adsorb water and let it slowly release water in bitumen. If the foam dissipation time can be prolonged and the bitumen expansion speed can be reduced, it will help to stabilize the performance of foamed bitumen. This paper conducts a study on the foaming characteristics and rheological properties of WPB based on waste molecular sieves. First, the bitumen foaming test is used to analyze the foaming characteristics of WPB with waste molecular sieves. Second, the basic properties of warm-mix foamed polymer-modified bitumen, including penetration, softening point, ductility, and viscosity, are investigated. Finally, a dynamic shear rheometer (DSR) is employed to study the high-temperature rutting resistance and high-temperature permanent deformation resistance of warm-mix foamed polymer-modified bitumen. The research results show that the amount of foaming water is the primary factor influencing bitumen foaming. The addition of waste molecular sieves has a significant impact on the intensity and duration of the bitumen foaming reaction. WPB with waste molecular sieves has a greater consistency and better high-temperature performance, but its low-temperature performance is somewhat weakened. The high-temperature deformation resistance of WPB with waste molecular sieves is superior to that of ordinary WPB and is affected by the amount of foaming water. An appropriate amount of foaming water can enable WPB with waste molecular sieves to exhibit excellent high-temperature deformation resistance.

## 1. Introduction

Bitumen pavement is made of bitumen mixtures formed by mixing bitumen and mineral aggregates. These mixtures are paved and compacted at high temperatures and can be formed into pavements of different thicknesses and types according to actual conditions [1]. Generally, bitumen mixtures are classified into two types, hot-mix and cold-mix bitumen mixtures, based on different preparation processes. Cold-mix bitumen mixtures are characterized by low energy consumption and low pollution and are currently well-recognized in the industry [2,3]. In contrast, the production of hot-mix bitumen mixtures requires a large amount of energy fuel, and a large amount of harmful gases are generated at high temperatures, which have a significant impact on the environment and the health of construction workers [4]. Therefore, many studies focus on how to reduce the mixing temperature of bitumen and how to control the emission of harmful gases from high-temperature bitumen.

In recent years, warm-mix foaming technology has become the mainstream research direction. By adding only 1–5% of water to the bitumen, the bitumen can be foamed, reducing the mixing temperature of the bitumen mixture. This not only reduces the energy consumption caused by high-temperature mixing but also has considerable environmental protection advantages [5,6,7,8]. The reduction in bitumen viscosity allows the bitumen to fully coat the aggregates, making the bitumen mixture more uniformly mixed and improving its workability. The control of bitumen foaming and water-stability factors are the weak technical areas in the promotion of WPB mixtures [9]. Poor foaming effects may lead to poor mixing and compaction results of the bitumen mixture, reduce the adhesion between bitumen and aggregates, and affect the pavement performance of the mixture [10,11].

Currently, scholars at home and abroad have conducted extensive research on foamed bitumen and achieved remarkable results. He et al. [12] studied the influence of foaming water dosage on the mechanical properties of foam warm-mix base bitumen mixtures and foam warm-mix SBS-modified bitumen mixtures. The study proposed the optimal water dosage thresholds of ≤1% for base bitumen and ≤2% for SBS-modified bitumen. Yahya et al. [13] investigated the effects of temperature and relative humidity on materials used in emulsion and foamed cement-bitumen treatments. The results showed that curing under a high temperature and low relative humidity yielded the highest mechanical properties, while an extended transition from low to high humidity reduced the performance. Cheng et al. [14] explored the relationship between the expansion ratio of foamed bitumen and its surface energy. The findings indicated a significant exponential relationship between the expansion ratio of individual bitumen bubbles and the surface energy of bitumen. Yu Xin et al. [11,15] studied the rheological properties of WPB and the influence of the amount of foaming water on these properties. The results indicated that the amount of water added has a great influence on the rheological properties of warm-mix foamed polymer-modified bitumen. An appropriate amount of foaming water can optimize the rheological properties of warm-mix foamed polymer-modified bitumen, and there is an interaction effect between different types of bitumen and water content. All the above studies focused on the influence of the amount of foaming water on bitumen foaming, but few studies have explored how to extend the foaming time and increase the foaming half-life [16,17,18].

Waste molecular sieves are mainly used in the production process of petrochemical products and have a catalytic effect. Due to their extremely fine pore-channel structures, they can be used for the foaming of WPB [19,20]. If waste molecular sieves are used as carriers for foaming water to prepare warm-mix foamed polymer-modified bitumen, and research and innovation are carried out on the warm-mix technology of warm-mix foamed polymer-modified bitumen, the disadvantages of high energy consumption and heavy pollution of hot-mix bitumen can be solved through effective improvement methods. At the same time, waste molecular sieves can be reused to address the problem of difficult disposal of waste molecular sieves, which conforms to the trend of the era of “resource conservation, green and low-carbon” [21,22,23].

In summary, although the existing research has clarified the influence of foaming water consumption, temperature, humidity, and other factors on the performance of foam warm-mix bitumen, it also proposed the optimal water threshold for different bitumen types and also revealed the correlation between the bitumen bubble expansion rate and surface energy. However, the multi-focus ‘immediate effect of foaming parameters on bitumen performance‘ lacks an in-depth exploration on how to extend the foaming time and improve the foaming half-life, which is a key technical bottleneck. It is difficult to fundamentally solve the problems of the poor mixing compaction effect and reduced adhesion caused by excessive foam dissipation. At the same time, the research on the application of porous materials such as waste molecular sieves as foaming water carriers in foam warm-mix bitumen has not been carried out, and the solid waste recycling has not been effectively combined with the optimization of foam warm-mix technology.

## 2. Research Scheme

**Figure 1:**
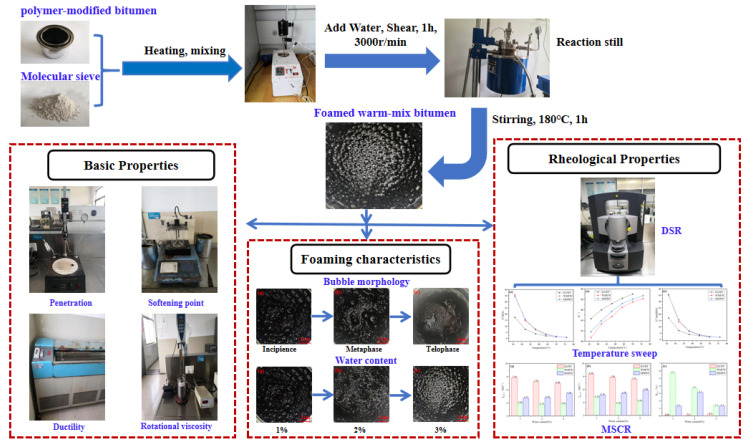
Flow chart of experimental planning.

Based on this, this paper aims to study the foaming characteristics and properties of WPB prepared with waste molecular sieves. First, the bitumen foaming test is used to analyze the foaming characteristics of WPB with waste molecular sieves, as well as the influence of the amount of foaming water and waste molecular sieves on the foaming characteristics. Second, the basic properties of WPB with waste molecular sieves, such as penetration, softening point, ductility, and viscosity, are studied. Finally, using a dynamic shear rheometer (DSR), through temperature sweep tests and multiple stress creep recovery (MSCR) tests, the high-temperature rutting resistance and high-temperature permanent deformation resistance of WPB with waste molecular sieves are investigated. The experimental planning process of this study is shown in Figure 1.

## 3. Material and Methods

### 3.1. Materials

#### 3.1.1. Polymer-Modified Bitumen

To address the issue of polymer-modified bitumen having high viscosity, which affects construction, this experiment prepared polymer-modified bitumen using waste polyurethane and base bitumen, with the waste polyurethane content set at 10%. The main technical properties of the bitumen were tested according to the relevant procedures in the “Test Regulations for bitumen and bitumen Mixtures in Highway Engineering” (JTG E20-2011) [24]. The results are shown in Table 1.

Polyurethane materials were obtained by crushing and processing waste plastic foam, with the particle size of polyurethane powder less than 0.075 mm. Polyurethane is a kind of soft foam, which comes from aromatic compounds. Properties of polyurethane are shown in Table 2.

#### 3.1.2. Waste Molecular Sieve

**Figure 2:**
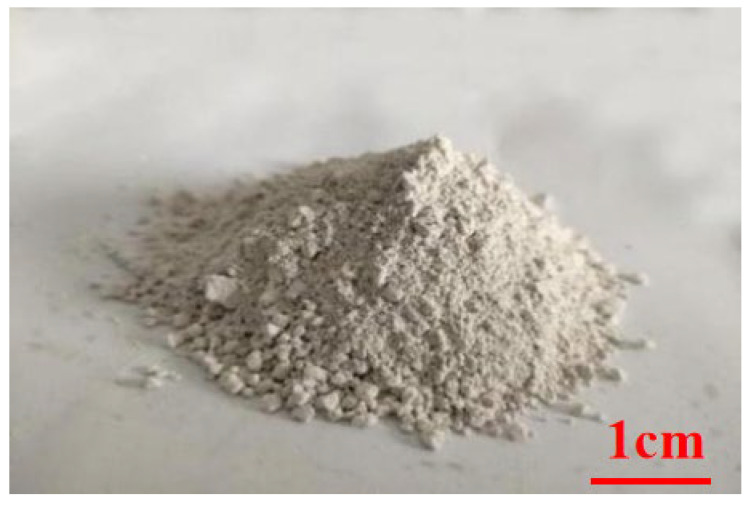
Industrial waste molecular sieve schematic diagram.

A molecular sieve is a kind of porous aluminosilicate material, according to the different proportions of silica and aluminum oxide and different pore structures. It has a nano-level microporous and mesoporous structure, and the pores are interconnected, so it can have a large specific surface area and pore volume. The uniform size distribution makes the molecular sieve have molecular selectivity, and it can separate molecules of different sizes, so it is called a molecular sieve. In this study, industrial waste molecular sieves were used, and the maximum water absorption was 83% [25]. The performance of the waste molecular sieve is shown in Table 3, and the shape is shown in Figure 2.

### 3.2. Preparation of WPB Based on Waste Molecular Sieve

#### 3.2.1. Preparation Test Design of WPB

The main purpose of this study is to use waste molecular sieves as carriers for bitumen foaming, and then study its foaming characteristics and bitumen properties, and conduct a comparative study with ordinary WPB. Therefore, in this study, a reaction kettle was used for foaming in the laboratory, and basic index tests were carried out.

To investigate the influence of the amount of foaming water and waste molecular sieves on bitumen foaming and its properties, this study designed 9 groups of comparative tests. The amount of base bitumen in each group was 600 g, and the bitumen foaming temperature was 145 °C. The first type is directly add water foaming (DAWF for short). The second type is waste molecular sieve foaming with water (WMFW for short). The third type is mix molecular sieve first and then add water to foam (MMWF for short). For these 3 groups of samples, 50 g of waste molecular sieves are first fully mixed with the base bitumen, and then 1% (6 g), 2% (12 g), and 3% (18 g) of water is added, respectively, for foaming. Meanwhile, during the sample preparation (bitumen foaming) process, the bitumen foaming state was observed, recorded (photographed), and the foaming characteristics were evaluated [26]. The mixture content is shown in Table 4.

#### 3.2.2. Preparation Process of WPB

**Figure 3:**
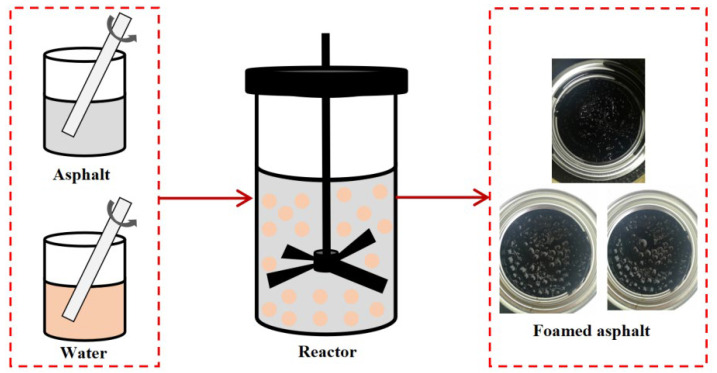
Preparation diagram of waste molecular sieve WPB.

The 600 g matrix bitumen was added to the reaction tank, heated, and stirred in a constant temperature reactor, and the temperature was set to 145 °C until the temperature of the matrix bitumen was stable, and the stirring was stopped. The mixture of water or waste molecular sieve and water was slowly added to the reaction tank, and the foaming characteristics were recorded. The preparation flow chart is shown in Figure 3. And ensure that the prepared foam warm-mix bitumen can be structurally stable before mixing with the aggregate.

### 3.3. Physical Properties Test

According to ASTMD36, ASTMD5, and ASTMD113 [24], the penetration (25 °C), softening point and ductility (5 °C) of waste molecular sieve WPB were tested. According to ASTMD4402, the viscosity of waste molecular sieve WPB at 135 °C was tested by rotating the viscosity equipment [27].

### 3.4. Rheological Properties Test

According to the ASTMD 7175 standard (AASHTO, 1995), a Kinexus dynamic shear rheometer was used to carry out a temperature scanning test on ordinary warm-mix foamed polymer-modified bitumen, waste molecular sieve WPB, and bitumen mixed with a waste molecular sieve before foaming. The diameter of the DSR plate is controlled to be 25 mm and the spacing is 1 mm. The frequency is fixed at 10 rad/s, and the experimental temperature range is 46–82 °C. The high-temperature performance indexes of bitumen measured by a 6 °C temperature interval are storage modulus (G′), loss modulus (G″), phase angle and rutting resistance factor G*/sin δ [28].

According to the ASTM D6648 and AASHTO T313 standards, the multiple stress creep recovery (MSCR) test was performed on a dynamic shear rheometer (DSR), and loading and unloading tests were performed under a stress control mode. At the temperature of 64 °C, the first loading 1 s under the stress of 0.1 kPa, unloading 9 s, is repeated 10 times. After that, the stress is increased to 3.2 kPa, and the above steps are repeated. The MSCR test mainly uses the unrecoverable creep compliance Jnr and the deformation recovery rate R to evaluate its performance [29].

## 4. Results and Discussion

Using the above materials and the experimental methods, the basic properties, foaming characteristics and rheological properties of foam polymer-modified bitumen were mainly studied. Based on the foaming characteristics and rheological properties of bitumen, the optimum content of molecular sieve and water in the preparation of foam bitumen were determined, so as to obtain a WPB with excellent foaming stability and popularity.

### 4.1. Physical Properties Analysis

#### 4.1.1. Basic Properties Analysis

**Figure 4:**
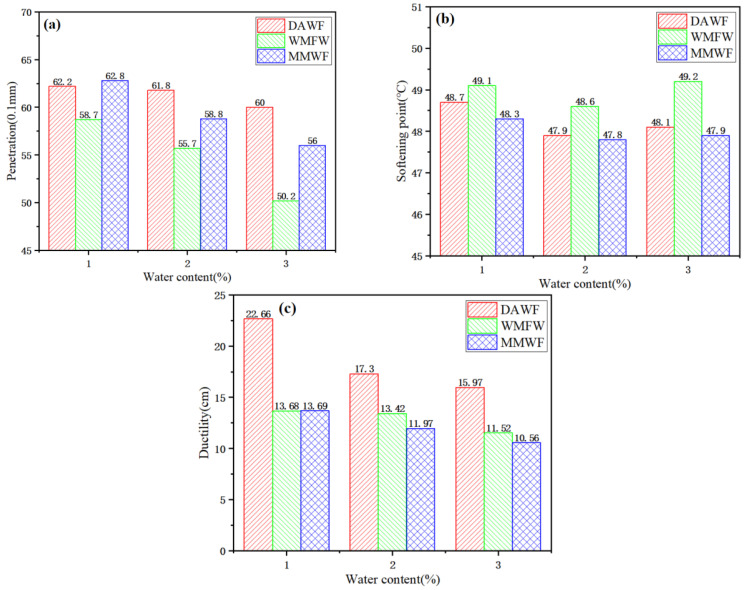
Basic properties of WPB, (**a**) Penetration, (**b**) Softening point, (**c**) Ductility.

As shown in Figure 4a, with the increase in the amount of added water, the 25 °C penetration of foamed bitumen for the three foaming methods is decreasing, indicating that the bitumen becomes harder. Under the condition of the same amount of added water, the penetration of bitumen that is foamed after adding water to waste molecular sieves first is the smallest. When the amount of added water is 1%, the penetration values of WPB by directly adding water and WPB after mixing with molecular sieves first are quite similar. When the amount of added water is 2% and 3%, the penetration of WPB by directly adding water is the largest, followed by the WPB after mixing with molecular sieves first and then adding water, and the smallest is the WPB after adding water to waste molecular sieves first. At the same time, it is found that although molecular sieves are added in all cases, due to different addition sequences and methods, the penetration of WPB is different in the end [30,31]. With the increase in water content, the penetration of bitumen in all three cases decreases. The range of decrease of the penetration of WPB by directly adding water is relatively small, while that of WPB after adding water to waste molecular sieves is relatively large.

As shown in Figure 4b, starting from a WAA of 1%, the softening points of WPB for the three foaming methods first decrease and then increase with the increase in the amount of added water. When the amount of added water is 1%, 2%, and 3%, the softening point of WPB after adding water to waste molecular sieves is the highest, indicating that this foaming method can improve the high-temperature performance of bitumen compared with the other two methods. The softening points of WPB for the three foaming methods all increase overall. With the increase in the amount of added water, the softening points first increase, then decrease, and then increase again. When the amount of added water is 1%, the increase range of the softening point of WPB after adding water to the waste molecular sieves is the largest. When the amount of added water increases from 2% to 3%, the increase range of the softening point of WPB after adding water to the waste molecular sieves is relatively large, while the increase ranges of the other two are relatively small, almost showing no change [32].

As shown in Figure 4c, with the increase in the amount of added water, the 10 °C ductility of WPB for the three foaming methods is decreasing, that is, the elastic performance becomes worse. The 10 °C ductility value of WPB by directly adding water is greater than that of the other two. Under the condition of the same amount of added water, the 10 °C ductility values of WPB for the two methods of foaming after adding water to waste molecular sieves and foaming after mixing waste molecular sieves first and then adding water are relatively close. The 10 °C ductility of WPB foamed by directly adding water is much larger than that of the other two, and with the increase in the amount of added water, the decreasing trend of the ductility of WPB becomes smaller. The change trends of the 10 °C ductility of WPB after adding water to waste molecular sieves and WPB after mixing waste molecular sieves first and then adding water are quite similar, and the ductility of WPB after adding water to waste molecular sieves is slightly larger.

#### 4.1.2. Viscosity Performance of Warm-Mix Foamed Polymer-Modified Bitumen

(1) Analysis of 145 °C viscosity change in warm-mix foamed polymer-modified bitumen

**Figure 5:**
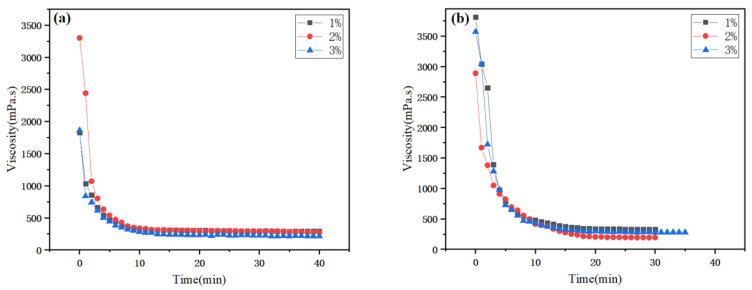
Viscosity variation in WPB with time, (**a**) DAWF, (**b**) WMFW.

Figure 5 is the 145 °C viscosity change diagram of WPB with direct water foaming and waste molecular sieve water foaming. The changes in bitumen from the beginning of foaming to viscosity stability under the two foaming methods are recorded. It can be seen from the figure that as the foaming progresses, the viscosity of the WPB begins to decrease sharply, then the decrease becomes smaller, and finally tends to be stable. The viscosity of WPB begins to stabilize at about 15 min, and the foaming of bitumen may be basically complete.

(2) Viscosity analysis of WPB at 135 °C, 145 °C and 175 °C

**Figure 6:**
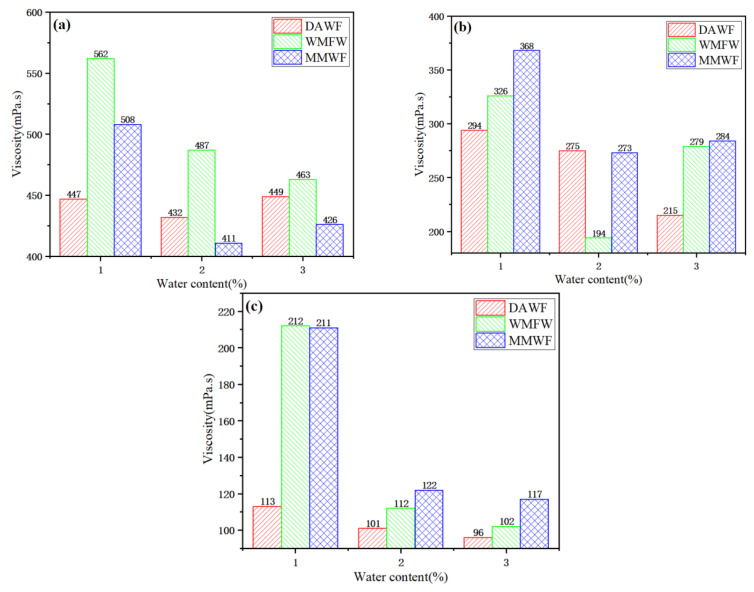
The change in viscosity–water content at different temperatures, (**a**) 135 °C, (**b**) 145 °C, and (**c**) 175 °C.

As shown in Figure 6a, the 135 °C viscosity of WPB with waste molecular sieves and added water for foaming is always greater than that of WPB foamed by the other two methods. The bitumen foamed by directly adding water has a relatively lower 135 °C viscosity. When the WAA is 3%, the 135 °C viscosity of WPB by directly adding water is slightly greater than that of WPB after mixing waste molecular sieves first and then adding water. This indicates that using waste molecular sieves as carriers for adding water and foaming bitumen can increase the 135 °C viscosity of WPB. It can also be seen that the decreasing trend of the 135 °C viscosity of WPB when the WAA increases from 1% to 2% is greater than that when the WAA increases from 2% to 3%.

As shown in Figure 6b, the 145 °C viscosity of WPB foamed by directly adding water is always the lowest, followed by that of WPB with waste molecular sieves and added water for foaming. The WPB after mixing waste molecular sieves first and then adding water has the highest 145 °C viscosity. Compared with directly adding water for foaming, using waste molecular sieves as carriers for adding water and foaming bitumen can also increase the 145 °C viscosity. It is also found that the change in the 145 °C viscosity of WPB foamed after mixing waste molecular sieves first and then adding water is relatively uniform with the increase in the WAA. When the WAA increases from 1% to 2%, the change trends of the 145 °C viscosity of WPB foamed by directly adding water and that with waste molecular sieves and added water for foaming are quite similar. However, when the WAA increases from 2% to 3%, the 145 °C viscosity of WPB foamed by directly adding water decreases more significantly, with a larger change trend.

As shown in Figure 6c, when the WAA is 1%, the 175 °C viscosity of WPB by directly adding water is much lower than that of the other two. With the increase in the WAA, the viscosities of WPB foamed by the three methods gradually approach each other. When the WAA is 3%, the viscosity differences among the three WPB are small. It can also be seen that the 175 °C viscosity of WPB foamed by directly adding water shows a small increasing trend with the increase in the WAA, but the change is not significant, indicating that the 175 °C viscosity of WPB of this foaming method is less affected by the WAA. For the other two foaming methods, when the WAA increases from 1% to 2%, the 175 °C viscosity of WPB decreases significantly with a large change trend. When the WAA increases from 2% to 3%, the change trend of the viscosity is small.

In conclusion, at the three temperatures, the viscosity of WPB with waste molecular sieves is greater than that of ordinary WPB. With the increase in the WAA, the viscosities of WPB at 135 °C, 145 °C, and 175 °C for the three foaming methods all decrease, but the decreasing amplitudes of viscosities at different temperatures vary. The viscosity of WPB is affected by the foaming method, that is, by waste molecular sieves. The addition of waste molecular sieves can increase the viscosity of bitumen.

(3) Effect of temperature on viscosity of WPB

**Figure 7:**
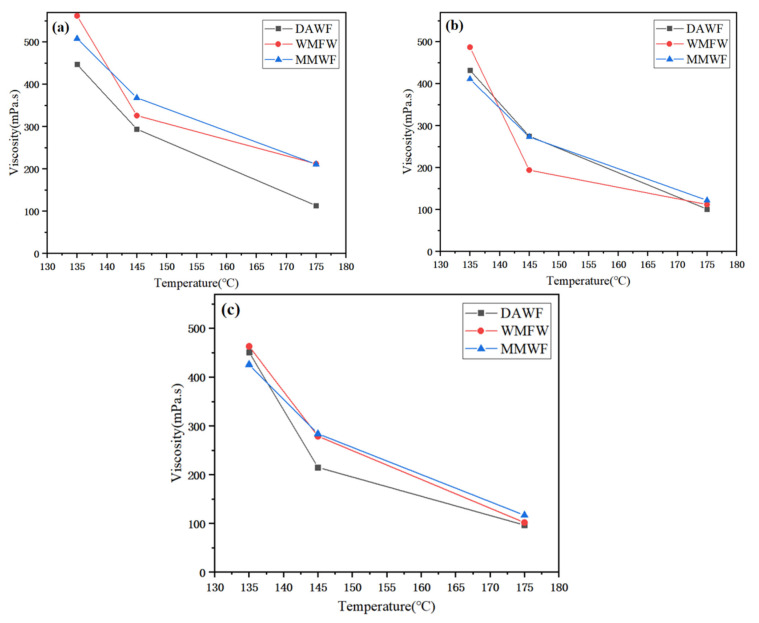
The temperature–viscosity diagram under different water content, (**a**) 1%, (**b**) 2%, and (**c**) 3%.

Figure 7a–c shows the viscosity–temperature change diagrams of the three foaming methods with WAA of 1%, 2%, and 3%. As can be seen from the figures, when the WAA is 1%, the viscosities of WPB foamed by directly adding water at three temperatures are all lower than those of WPB by the other two methods. With the increase in temperature, the viscosities of WPB continuously decrease, and the decreasing amplitude also becomes smaller. The viscosity changes in WPB for the two methods of foaming with waste molecular sieves and added water and foaming after mixing waste molecular sieves with bitumen first are quite similar [33]. When the WAA is 2%, the viscosities of WPB for the three foaming methods all decrease with the increase in temperature, and the decreasing amplitudes are comparable. The viscosity values of WPB by the three methods at 135 °C and 175 °C are relatively close, and the 145 °C viscosity of WPB foamed by directly adding water is lower than that of the other two.

In conclusion, at the same temperature, the viscosities of WPB with different foaming methods are different. With the increase in temperature, the viscosities of WPB for the three foaming methods with different WAA all decrease, and generally, the decreasing amplitudes are large first and then small.

### 4.2. Study on Foaming Characteristics of WPB

**Figure 8:**
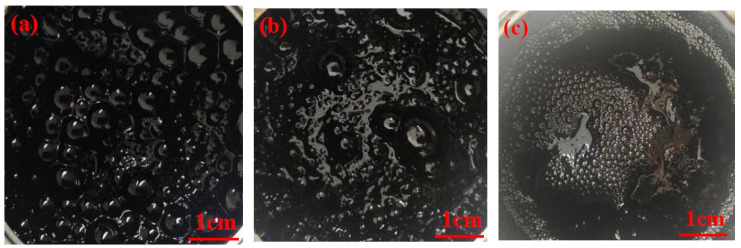
Foaming form of WPB, (**a**) incipience, (**b**) metaphase, and (**c**) telophase.

During the bitumen foaming process, a shooting device was used to capture the morphology of bitumen foam at the early, middle (10 min), and late (2 h) stages of bitumen foaming. The foaming characteristics of waste molecular sieves were evaluated mainly by observing the intuitive pictures of bitumen foaming (the amount of foam, the size of foam, and the dissipation time of foam). As shown in Figure 8, during the bitumen foaming process, as the water evaporates, the volume of WPB will gradually increase until it reaches a peak. After a few seconds, the volume of WPB will rapidly decrease. In fact, the change in volume is due to the change in bitumen viscosity. When the volume increases, the viscosity of bitumen decreases. In the initial stage of bitumen foaming, there are many large and unstable foams in the WPB. As the foaming progresses, the foams will gradually decrease in size and quantity. At the end of the foaming process, the foams in the WPB are small and stable. It was also found that during the bitumen foaming process, the foams gradually changed from polyhedrons to spheres and finally burst. Figure 8 shows the morphological diagrams of bitumen at the initial, middle, and final stages of foaming.

**Figure 9:**
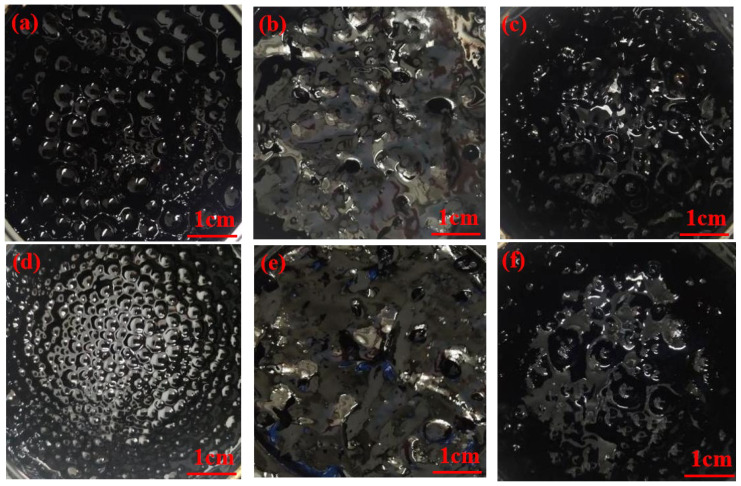
The initial morphology of WPB by three foaming methods, (**a**) DAWF + 1%, (**b**) WMFW + 1%, (**c**) MMWF + 1%, (**d**) DAWF + 3%, (**e**) WMFW + 3%, and (**f**) MMWF + 3%.

**Figure 10:**
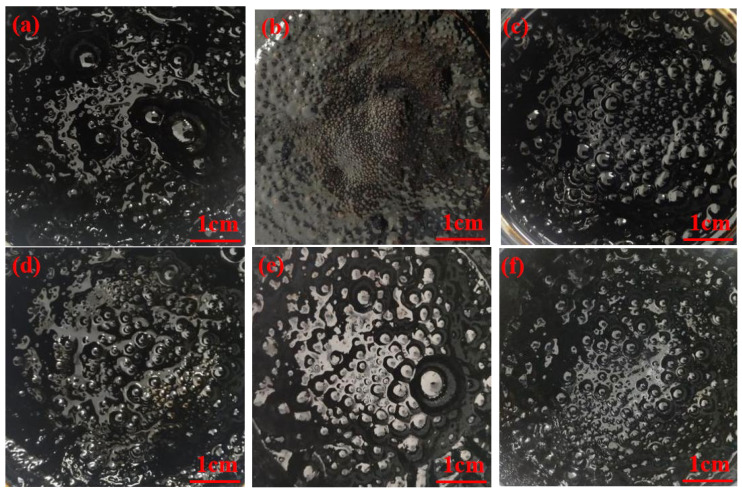
The morphology of WPB in the middle stage of three foaming methods, (**a**) DAWF + 1%, (**b**) WMFW + 1%, (**c**) MMWF + 1%, (**d**) DAWF + 3%, (**e**) WMFW + 3%, and (**f**) MMWF + 3%.

In order to study the effect of waste molecular sieve on the foaming characteristics of bitumen, the bitumen morphology of three foaming methods, such as direct mixing water foaming, waste molecular sieve mixing water foaming, and bitumen mixing waste molecular sieve first and then mixing water foaming, was compared and observed. This section discusses the foam morphology of the three foaming methods in the early and middle stages of foaming with 1% and 3% water (foaming for 10 min). Figure 9 and Figure 10 are the bitumen foam morphology diagrams of the three foaming methods.

As shown in Figure 9, from the intuitive representation of the morphological diagrams of bitumen in the initial stage of foaming, in the initial stage of directly adding water for foaming, there are many bitumen foams with clear bubble outlines, and the bitumen appears clear and translucent. For the bitumen that is first mixed with waste molecular sieves and then foamed, the foam is slightly thick, with a small number of visible bubble-like foams that are uneven in size and in a chaotic state. The bitumen that is foamed after adding water to waste molecular sieves is relatively thick, with almost no visible bubble-like foams. In the initial stage of the reaction, the reaction of foaming with waste molecular sieves and added water is the most intense. Bubbles are generated during the reaction but burst quickly, accompanied by a dull popping sound of bubbles. Therefore, there are almost no visible bubbles in the early stage of the reaction. Next is the bitumen that is first mixed with waste molecular sieves and then foamed. The reaction is also relatively intense, with a loud popping sound of bubbles. Although the bubbles do not burst as quickly as those in the case of foaming with waste molecular sieves and added water, most of the bubbles still burst quickly. The reaction of directly adding water for foaming is also quite intense, but it is a bit less intense compared with the other two methods. A large number of bubbles are generated during the reaction, accompanied by a clear popping sound of bubbles, and the bubbles continue to be produced.

As shown in Figure 10, when the WPB with directly added water reaches the middle stage (10 min) of the reaction, the numbers of bitumen foams decrease and the bubbles become smaller. For the WPB with waste molecular sieves and added water in the middle stage of the reaction, compared with the start of the reaction, a small number of foams with clear outlines appear, and the foams with a higher WAA are more obvious. In the middle stage of the reaction of WPB with waste molecular sieves first mixed and then water added for foaming, a large number of bubbles with obvious outlines appear. Judging from the actual foaming situation, when the WPB with directly added water reaches the middle stage of the reaction, the intensity of the reaction is relatively low, the rate of foam generation decreases, and the reaction duration is short. For the WPB foamed by the other two methods, the intensity of the reaction is greatly reduced, and the reaction duration in the later stage is longer than that of directly adding water for foaming. The reaction of WPB with waste molecular sieves and added water is slow in the later stage but has a very long duration [30].

In conclusion, the addition of waste molecular sieves has a significant impact on the foaming reaction, mainly reflected in the state of the WPB at each stage of the reaction and the reaction duration. Meanwhile, the way of adding waste molecular sieves also has an impact. Compared with the WPB with directly added water, the WPB with waste molecular sieves and added water has a larger expansion rate in the early stage of the reaction and a longer reaction time.

### 4.3. Rheological Properties of Waste Molecular Sieve WPB

#### 4.3.1. Effect of Waste Molecular Sieve on High-Temperature Anti-Rutting Performance of WPB

**Figure 11:**
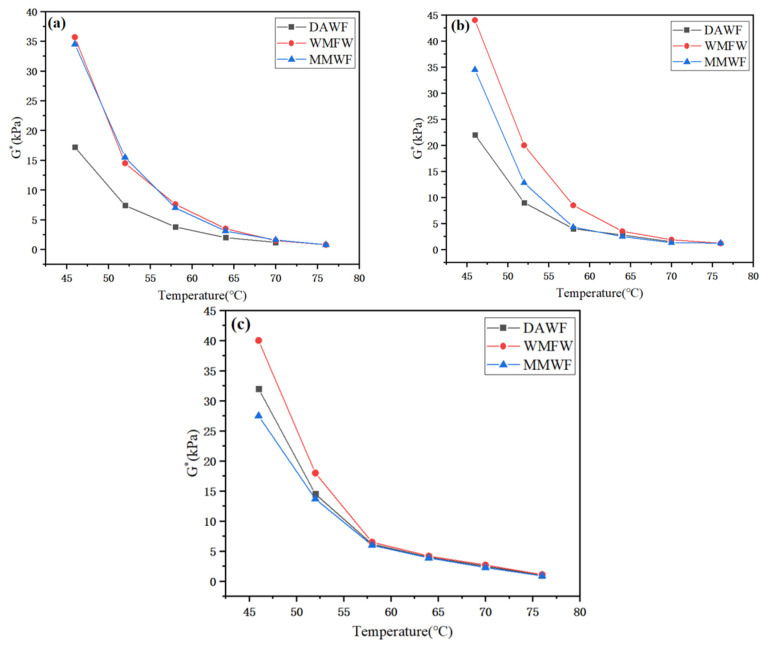
Complex shear modulus of WPB with different water contents, (**a**) 1%, (**b**) 2%, and (**c**) 3%.

**Figure 12:**
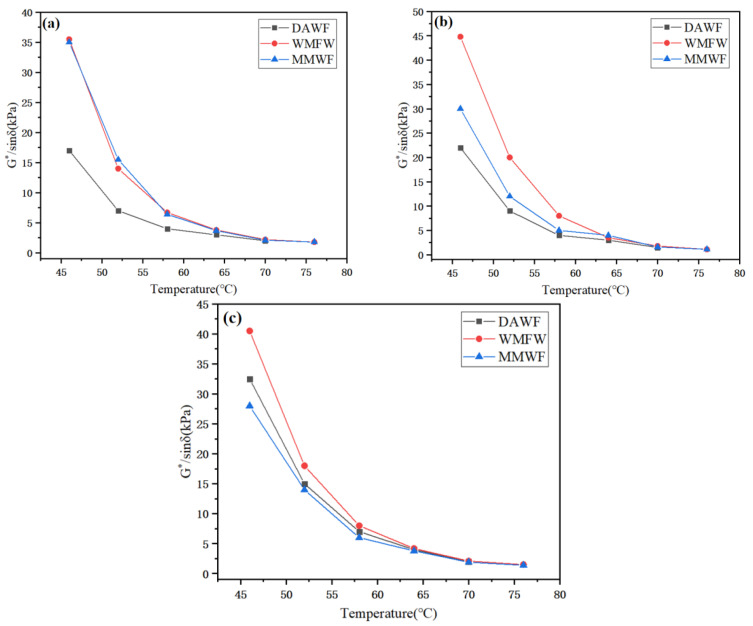
Anti-rutting factor of WPB with different water contents, (**a**) 1%, (**b**) 2%, and (**c**) 3%.

As can be seen from Figure 11 and Figure 12, the change trends of the complex shear modulus G* and the rutting factor G*/sin δ of ordinary WPB, WPB with waste molecular sieves, and WPB after mixing with waste molecular sieves are the same. Their values decrease with the increase in temperature, indicating that the deformation resistance of the three types of bitumen weakens as the temperature rises. At a different WAA and any temperature, the complex shear modulus G* and the rutting factor G*/sin δ of ordinary WPB are smaller than those of WPB by the other two methods. When the WAA is 1%, the values and change trends of WPB with waste molecular sieves and WPB foamed after mixing waste molecular sieves with bitumen first are relatively close. When the WAA is 2% and 3%, the values of WPB with waste molecular sieves at various temperatures are relatively large. This shows that the addition of waste molecular sieves can improve the high-temperature deformation resistance of WPB, and using waste molecular sieves as a foaming carrier, mixing them with water first and then foaming can further enhance the deformation resistance of WPB. According to the SHRP specification, the rutting factor of bitumen G*/sin δ ≥ 1.0 kPa, and the temperature corresponding to this critical value is the critical temperature. The higher the critical temperature specified in the specification, the stronger the high-temperature rutting resistance of the bitumen, and vice versa. In the experiment, the critical temperatures of ordinary WPB, WPB with waste molecular sieves, and WPB foamed after mixing waste molecular sieves with bitumen first were measured to be 67.3 °C, 73.3 °C, and 72.8 °C, respectively, when the WAA was 1%; 68.1 °C, 75.2 °C, and 71.4 °C when the WAA was 2%; and 72.9 °C, 73.6 °C, and 71.3 °C when the WAA was 3%. It can be seen that WPB with waste molecular sieves has better high-temperature performance.

**Figure 13:**
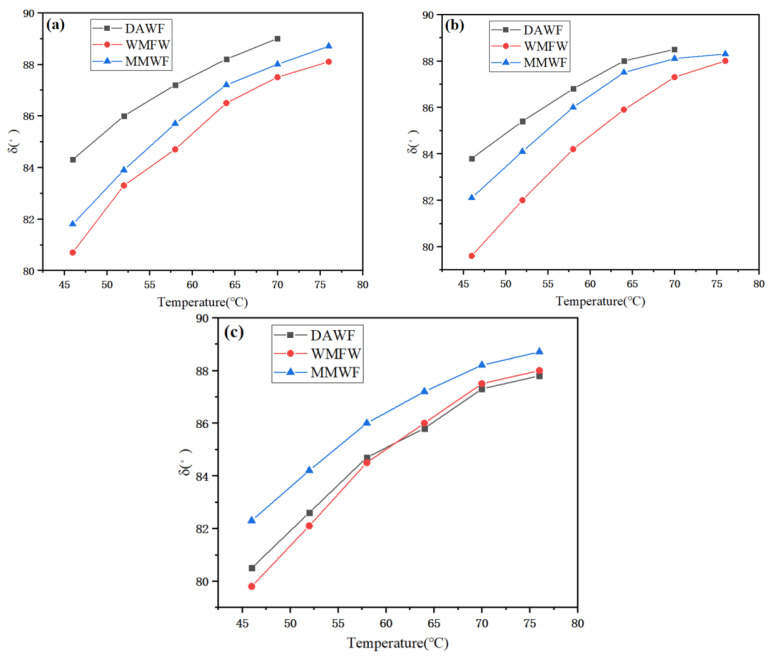
Phase angle of WPB with different water contents, (**a**) 1%, (**b**) 2%, and (**c**) 3%.

As can be seen from Figure 13, the phase angles δ of WPB foamed by the three methods all increase with the increase in temperature, indicating that as the temperature rises, the viscosity of WPB increases while the elasticity decreases. When the foaming WAA are 1% and 2%, at the same temperature, the phase-angle magnitudes of the three types of WPB are as follows: WPB with waste molecular sieves < WPB foamed after mixing waste molecular sieves with bitumen first < ordinary WPB. The main reason is that the addition of waste molecular sieves enhances the elastic properties of WPB, and the addition method also affects the elasticity of WPB. That is, the elastic properties of WPB with waste molecular sieves are better. When the foaming WAA is 3%, the relationship between the phase-angle δ values of the three types of WPB changes significantly. At this time, at each temperature, the phase-angle δ value of WPB foamed after mixing waste molecular sieves with bitumen first is greater than that of the other two. When the temperature is lower than 59.6 °C, the phase angle of ordinary WPB is smaller than that of WPB with waste molecular sieves. When the temperature is higher than 59.6 °C, the phase angle of ordinary WPB is greater than that of WPB with waste molecular sieves. The reason for this change may be related to the amount of waste molecular sieves, the amount of added water, and the mechanism of waste molecular sieves in the bitumen foaming process.

#### 4.3.2. High-Temperature Resistance to Permanent Deformation Performance of WPB

**Figure 14:**
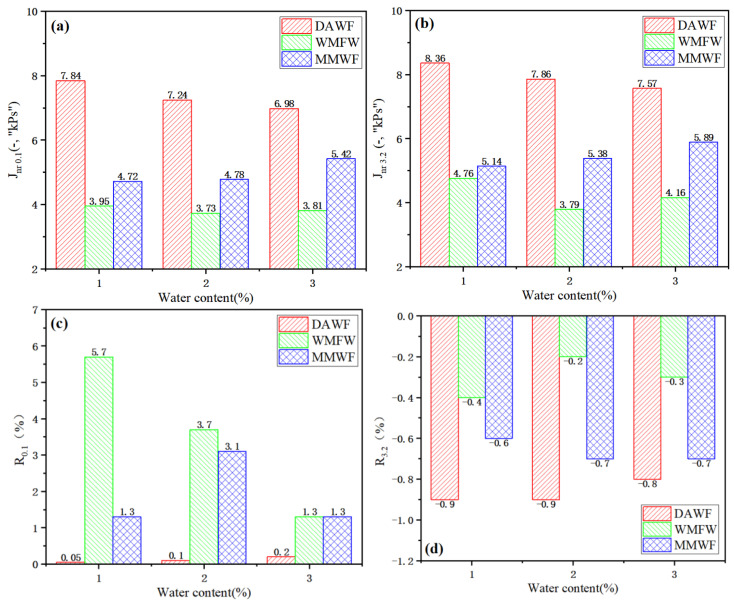
MSCR test of WPB with three foaming methods, (**a**) Jnr_0.1_, (**b**) Jnr_3.2_, (**c**) R_0.1_, (**d**) R_3.2_.

Based on the MSCR test, the high-temperature permanent deformation resistance of WPB with waste molecular sieves was studied. The MSCR test temperature selected in this paper was 64 °C, and the test results are shown in Figure 14. For the MSCR test indicators, the smaller the value of the non-recoverable creep compliance Jnr, the smaller the non-recoverable deformation of the bitumen, and the stronger the deformation resistance under high-temperature conditions, conversely, the weaker it is. The larger the deformation recovery rate R, the stronger the elastic deformation ability of the bitumen, conversely, the weaker it is.

As can be seen from Figure 14, when the amount of foaming water is the same, the values of Jnr_0.1_ and Jnr_3.2_ of the three types of WPB show that those of WPB with waste molecular sieves are the smallest, and those of ordinary WPB are the largest, indicating that the high-temperature performance of WPB with waste molecular sieves is better. With the increase in the amount of added water, the values of Jnr_0.1_ and Jnr_3.2_ of ordinary WPB show a decreasing trend, indicating that within the range of the foaming water amount in the test, the larger the amount of water, the better the high-temperature performance of ordinary WPB. The values of Jnr_0.1_ and Jnr_3.2_ of the WPB after mixing waste molecular sieves first increase with the increase in the amount of foaming water, indicating that the high-temperature performance of this WPB weakens. The values of Jnr_0.1_ and Jnr_3.2_ of WPB with waste molecular sieves first decrease and then increase with the increase in the amount of added water, indicating that the high-temperature performance of WPB with waste molecular sieves is better when the amount of added water is 2% [16].

In terms of the deformation recovery rate R, the R value of ordinary WPB is very small, and the bitumen basically exhibits viscous characteristics. When the amount of added water is the same, the R values of the three types of WPB show that those of WPB with waste molecular sieves are the largest, and those of ordinary WPB are the smallest, indicating that WPB with waste molecular sieves has better elastic performance and stronger deformation resistance, which is consistent with the law analyzed by Jnr.

In conclusion, the high-temperature permanent deformation resistance of WPB with waste molecular sieves is better than that of ordinary WPB, and it is affected by the amount of foaming water. An appropriate amount of foaming water can enable WPB with waste molecular sieves to exhibit excellent high-temperature deformation resistance.

## 5. Conclusions

This paper studies the foaming characteristics of WPB with waste molecular sieves and the properties of WPB with waste molecular sieves, and the following main conclusions are drawn:

(1) The amount of foaming water is a major factor affecting bitumen foaming. As the amount of foaming water added to bitumen increases, the intensity of the bitumen reaction becomes greater, the amount of foam increases in the early stage of the reaction, the expansion ratio becomes larger, and the reaction duration is longer.
(2) The addition of waste molecular sieves has a significant impact on bitumen foaming. Waste molecular sieves slow down the bitumen foaming reaction, making the bitumen appear viscous during the reaction, reducing the expansion ratio and the amount of foam in the early stage of the reaction, while prolonging the reaction duration. Among them, when bitumen is foamed by means of waste molecular sieves mixed with water, the reaction duration of the foamed bitumen is longer.
(3) The penetration of WPB with waste molecular sieves is slightly smaller than that of ordinary WPB, the softening point is slightly higher than that of ordinary WPB, and the 10 °C ductility is slightly smaller than that of ordinary bitumen. The viscosity of WPB with waste molecular sieves is affected by the amount of foaming water and temperature. The viscosities of WPB with waste molecular sieves at 135 °C, 145 °C, and 175 °C are all greater than those of ordinary WPB. The high-temperature performance of WPB with waste molecular sieves is improved, while the low-temperature performance is weakened.
(4) The high-temperature deformation resistance of waste molecular sieve foamed bitumen is superior to that of ordinary foamed bitumen, and it is influenced by the amount of foaming water. An appropriate amount of foaming water enables waste molecular sieve foamed bitumen to exhibit excellent high-temperature deformation resistance.
(5) When preparing foam warm-mix polymer-modified bitumen, the waste molecular sieve is first allowed to absorb water and is then added to the high-temperature polymer-modified bitumen to foam. The prepared bitumen has the best performance and the best foaming stability. At the same time, the optimum water consumption in the foaming process of foamed bitumen is 2%. Therefore, the foamed bitumen can be prepared by using waste molecular sieve to absorb 2% water and directly put into foaming.

This paper only studies the foaming characteristics and rheological properties of foamed bitumen. Research on the mechanism of molecular sieves in the foaming process will help improve various properties of waste molecular sieve foamed bitumen by adjusting certain factors from the perspective of the reaction process. Meanwhile, future research should be extended to the performance of waste molecular sieve foamed bitumen mixtures, such as the high-temperature performance stability, low-temperature crack resistance, and water stability of bitumen mixtures. The study of these properties will facilitate a better evaluation of the road performance of waste molecular sieve foamed bitumen mixtures.

## Figures and Tables

**Table 1:** Properties of bitumen.

Parameter	Unit	Results	Standard (JTG E20-2011) [24]
Penetration (25 °C)	0.1 mm	59.7	T0604
Softening point	°C	80.2	T0606
Ductility (5 °C)	cm	33.8	T0605
Dynamic viscosity (60 °C)	Pa.s	283.2	T0620
Density (15 °C)	g·cm^−3^	1.06	T0603
Flash point	°C	463	T0611

**Table 2:** Properties of polyurethane.

Parameter	Results	Standards
Density (g/cm3)	1.16	DIN 53479
Tensile strength (MPa)	42	DIN 53504-S2
Tear strength (kN/m)	0.064	DIN 53515
Softening point (°C)	71	ISO 306

**Table 3:** Properties of waste molecular sieves.

Property	Requirements	Results
Static water absorption/%	≥21	43
Actual aperture	—	9A
Packing density (g/cm^3^)	0.70–0.75	0.74
Abrasion ratio/wt%	≤0.3	0.17
Al_2_O_3_ content/%	≥92	93.6
SiO_2_ content/%	≤0.3	0.014
Fe_2_O_3_ content/%	≤0.03	0.011
Na_2_O content/%	≤0.5	0.243

**Table 4:** The yield of each mixture component.

Specimen	Bitumen/%	Polyurethane/%	Molecular Sieve/%	Water/%
Category I	100	10	0	2001/2/3
Category II	100	10	8.3	2001/2/3
Category III	100	10	8.3	2001/2/3

## Data Availability

The datasets used and/or analyzed during the current study are available from the corresponding author on reasonable request.
